# High-Endurance Long-Term Potentiation in Neuromorphic Organic Electrochemical Transistors by PEDOT:PSS Electrochemical Polymerization on the Gate Electrode

**DOI:** 10.1021/acsami.3c10576

**Published:** 2023-11-15

**Authors:** Federica Mariani, Francesco Decataldo, Filippo Bonafè, Marta Tessarolo, Tobias Cramer, Isacco Gualandi, Beatrice Fraboni, Erika Scavetta

**Affiliations:** † Department of Industrial Chemistry “Toso Montanari”, Alma Mater Studiorum - University of Bologna, Viale del Risorgimento 4, 40136 Bologna, Italy; ‡ Department of Physics and Astronomy, Alma Mater Studiorum - University of Bologna, Viale Berti Pichat 6/2, 40127 Bologna, Italy

**Keywords:** organic electrochemical transistor, neuromorphic device, long-term potentiation, synaptic plasticity, paired-pulse depression, electrodeposition

## Abstract

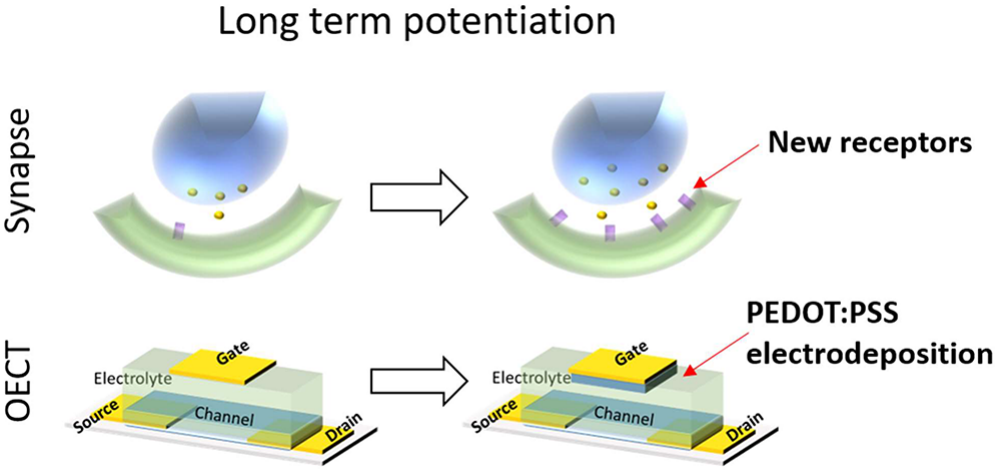

The brain exhibits extraordinary information processing capabilities thanks to neural networks that can operate in parallel with minimal energy consumption. Memory and learning require the creation of new neural networks through the long-term modification of the structure of the synapses, a phenomenon called long-term plasticity. Here, we use an organic electrochemical transistor to simulate long-term potentiation and depotentiation processes. Similarly to what happens in a synapse, the polymerization of the 3,4-ethylenedioxythiophene (EDOT) on the gate electrode modifies the structure of the device and boosts the ability of the gate potential to modify the conductivity of the channel. Operando AFM measurements were carried out to demonstrate the correlation between neuromorphic behavior and modification of the gate electrode. Long-term enhancement depends on both the number of pulses used and the gate potential, which generates long-term potentiation when a threshold of +0.7 V is overcome. Long-term depotentiation occurs by applying a +3.0 V potential and exploits the overoxidation of the deposited PEDOT:PSS. The induced states are stable for at least 2 months. The developed device shows very interesting characteristics in the field of neuromorphic electronics.

## Introduction

Artificial intelligence and deep learning algorithms are important emerging and enabling tecnhologies employed in different commercial products and services such as web searching^1^ and language understanding.^2^ The main applications are at the software level and are executed on computers based on the conventional von Neumann architecture, which operates sequentially and does not emulate the brain, which works in a massively parallel fashion through a densely interconnected network of neurons.^3^ Research efforts have been devoted to the development of neuromorphic devices that simulate the synapsis operation at a hardware level to fill the gap between electronics and the human brain.^4−6^ Neuromorphic features have been obtained by exploiting capacitors, transistors, and memristors. Organic electrochemical transistors (OECTs) are gaining momentum in the recent literature among neuromorphic devices.^4,7^

OECTs are attracting growing interest in the field of artificial intelligence, as they are able to emulate numerous biological processes with hardware components, such as short-term plasticity,^8,9^ spike-dependent plasticity,^8,10^ and homoestatic plasticity.^11,12^ While short-term processes can be successfully simulated, further research efforts are needed to increase the retention of the induced states in long-term potentiation that are traditionally generated through a change in the redox state of the conducting polymer constituting the transistor channel. Since the reduced/oxidized conductive polymer slowly comes back to its pristine state, the neuromorphic effect remains stable only for a few hours.^10,13−15^ A recent successful approach to increase the retention time in OECT neuromorphic devices is based on electrodeposition occurring between the source and drain terminals to form the channel in a way that is similar to memristors based on a localized conductive filament.^16,17^ Following these few successful examples, the use of electropolymerization in neuromorphic devices needs to be further explored to identify new approaches that could help the implementation of high-endurance functionalities in artificial intelligence hardware systems.

The solid structure of an OECT is composed by a stripe of conductive polymer that works as a channel and by another electrode that works as a gate.^15,18^ A proper electrolyte completes the device by ionically connecting the channel to the gate. The voltage applied to the gate electrode modulates the current flowing in the channel due to the stimulation of electrochemical reactions involving the conductive polymer. These processes involve the ions, which must diffuse inside the semiconductor material to keep the electroneutrality. In neuromorphic OECTs, the most used correspondence between synapses and transistors assigns the presynaptic signal to the gate voltage, because it acts as a switch and controls the conductivity of the channel. Similarly to the action potential in the synapse, the voltage applied to the gate regulates the current flowing in the channel, which represents the postsynaptic signal. The synaptic weight is instead associated with the conductance variation assumed by the channel that can be calculated as transconductance. OECTs are able to read and receive stimuli simultaneously, as it is also possible to fabricate devices with multiple gates, which allow the reproduction of spatiotemporal effects typical of synapses.^19^ Other very important characteristics are (i) efficient transduction of ionic-electronic currents typical of synapses;^20^ (ii) ability to operate in the electrolytic or biological environment necessary for future use in the biomedical field;^18^ (iii) possibility of integration in flexible substrates;^12^ (iv) low absorbed power. Short-term plasticity in OECTs has been induced by exploiting the transistor ionic circuit and the relatively high relaxation time required for the ions for entering inside or exiting from the conductive polymer.

The long-term potentiation is usually induced by varying the redox state of the conductive polymer. This is achieved by applying a proper *V*_g_ signal for a short time to an otherwise floating gate electrode whose switch exhibits an energy cost. The state-retention time, which is the duration of the neuromorphic effect, plays a key role for this neuromorphic behavior. The requirement for this parameter can vary significantly depending on the application, but generally, longer state-retention times are desired. Moreover, the discrimination of STP and LTP phenomena is usually based on the duration of the neuromorphic behavior for neuromorphic electronics. STP disappears within a few seconds, while LTP refers to changes that persist for tens of seconds or longer.^21^ Similarly to conventional memories, the information is memorized as a variation of conductivity of the channel, and thus, the training algoritm is performed thanks to the applied *V*_g_, which cannot be used during the reading procedure. Nevertheless, the conductive polymer slowly comes back to its pristine redox state due to the electrochemical reactions with ubiquitous compounds such as oxygen. Salleo’s group devoted research effort to improve the retention time in PEDOT:PSS by combining the polymer with a compound able to stabilize the induced redox state (i.e., polyethylenimine) or by encapsulating the device to hinder unwanted reactions.^10^ Despite these attempts, the endurance of long-term potentiation is limited to a few days. Electropolymerization is a fascinating way to overcome these limitations. It offers the potential to achieve retention in artificial synapses by real morphological changes instead of volatile electrical ones, thus emulating more closely the unique ability of the brain to make new connections where none existed before. Gerasimov et al. exploited the electrodeposition of poly(sodium 4-(2-(2,5-bis(2,3-dihydrothieno[3,4-*b*][1,4]dioxin-5-yl)thiophen-3-yl)ethoxy)butane-1-sulfonate) (PETE-S) to create conductive pathways between the source and drain electrodes to fabricate an evolvable neuromorphic OECT.^17^ The new connection is stable for months. Similarly, Janzakova et al. exploited the electropolymerization of 3D PEDOT:PSS networks for generating a dendritic connection that costituites the channel of the OECT.^16^ All these approaches are based on the variation of channel conductance that is used as a memory element in the neuromorphic device. The gate voltage is used as a stimulus in the training cycles, but it plays no role in the reading phase because the devices operate without applying a potential to the gate. Therefore, the gate voltage loses its role of action potential in the standard operation of the neuromorphic device. Moreover, it is worthy to note that long-term potentiation can take place in nature by modification occurring at both presynaptic and postsynaptic neurons.^22,23^ Therefore, new ways should be explored to increase the approach for obtaining specific abilities in neuromorphic devices.

Here, we have explored the opportunity of electrodepositing PEDOT:PSS on the gate electrode to obtain long-term potentiation for the first time. Differently from other approaches based on electrosynthesis reported in the literature, the neuromorphic behavior does not arise from the channel as a memory element but modifies the gating efficiency of the OECT and so its switching properties. Thanks to its volumetric capacitance, the deposition of PEDOT:PSS film increases the gate capacitance and thus the transistor transcondutance, i.e., the ability of the gate electrode in modulating the current flowing in the channel. As happens in synapses, the long-term potentiation is the result of a structural change of the devices, which strengthens the connection between the presynaptic elements (ideally the gate) and the postsynaptic element (ideally the channel). The phenomena have been systematically investigated to identify the effect of gate voltage (i.e., presynaptic signal) on the learning ability of our device. Finally, we have verified that the neurorphic device exhibits short-term plasiticity as already demonstrated for other OECTs.

## Experimental Section

### Reagents and Instruments

Clevios PH1000 was purchased from Heraeus. Ethylene glycol (EG), dodecyl benzenesulfonic acid (DBSA), 3-glycidoxypropyl trimethoxysilane (GOPS), EDOT monomer, and PSS:Na were purchased from Sigma-Aldrich. Microposit S1818 positive photoresist, Microposit MF-319 developer, mr-DWL 5 negative photoresist, and mr-DWL 5 negative photoresist were purchased from Micro Resist Technology. Device fabrication through lithography was performed using an ML3Microwriter from Durham Magneto Optics. Neuromorphic experiments were carried out with a Source-measure Unit (Keysight B2902A). In situ AFM measurements were performed using a Park NX10 atomic force microscope with a PPP-NCHR cantilever (force constant, 34.55 N/m). The impedance spectra of the gate electrode after the long-term potentiation procedure were acquired using a MFLI lock-in amplifier (from Zurich Instruments).

### Device Fabrication

Glass substrates were cleaned by sonication in water and soap (10%)/acetone/isopropanol/distilled water baths. After the dehydration step (10 min at 110 °C), the Microposit S1818 positive photoresist (from Micro Resist Technology) was spin coated (4000 rpm for 60 s) and annealed at 110 °C for 1 min. Metallic contacts were patterned through direct laser lithography using the ML3Microwriter (from Durham Magneto Optics). The photoresist was developed with a Microposit MF-319 developer. Then, 7 nm of chromium and 30 nm of gold were deposited by thermal evaporation. Samples were immersed in acetone for 4 h for photoresist liftoff. Metallic contacts were encapsulated using an mr-DWL 5 negative photoresist (from Micro Resist Technology). The resin was spin coated at 3000 rpm for 30 s and annealed at 100 °C for 2 min. After laser exposure, samples were baked at 100 °C for 2 min, relaxed for 1 h at room temperature, and developed with mr-Dev 600 developer (Micro Resist Technology). A final oxygen plasma descum of photoresist residuals (120 W for 4 min) was performed, and then, the negative resist was baked at 120 °C for 30 min. A double layer of S1818 was deposited to pattern the PEDOT:PSS microstructures (the OECT channel). After development, substrates were treated with air plasma (15 W for 2 min), and the PEDOT:PSS solution (94% PEDOT:PSS (Heraeus, Clevios PH1000), 5% of ethylene glycol (EG) (Sigma-Aldrich), 1% of 3-glycidoxypropyltrimethoxysilane (GOPS), and 0.25% of 4-dodecylbenzenesulfonicacid (DBSA)) was spin coated at 3000 rpm for 10 s. The resulting film thickness was (100 ± 10) nm. The samples were subsequently annealed at 120 °C for 1 h, and S1818 was finally lifted off after 4 h in acetone. The neuromorphic OECT works in a PSS:Na solution containing 10 mM EDOT.

### Long-Term Plasticity

**Figure 1:**
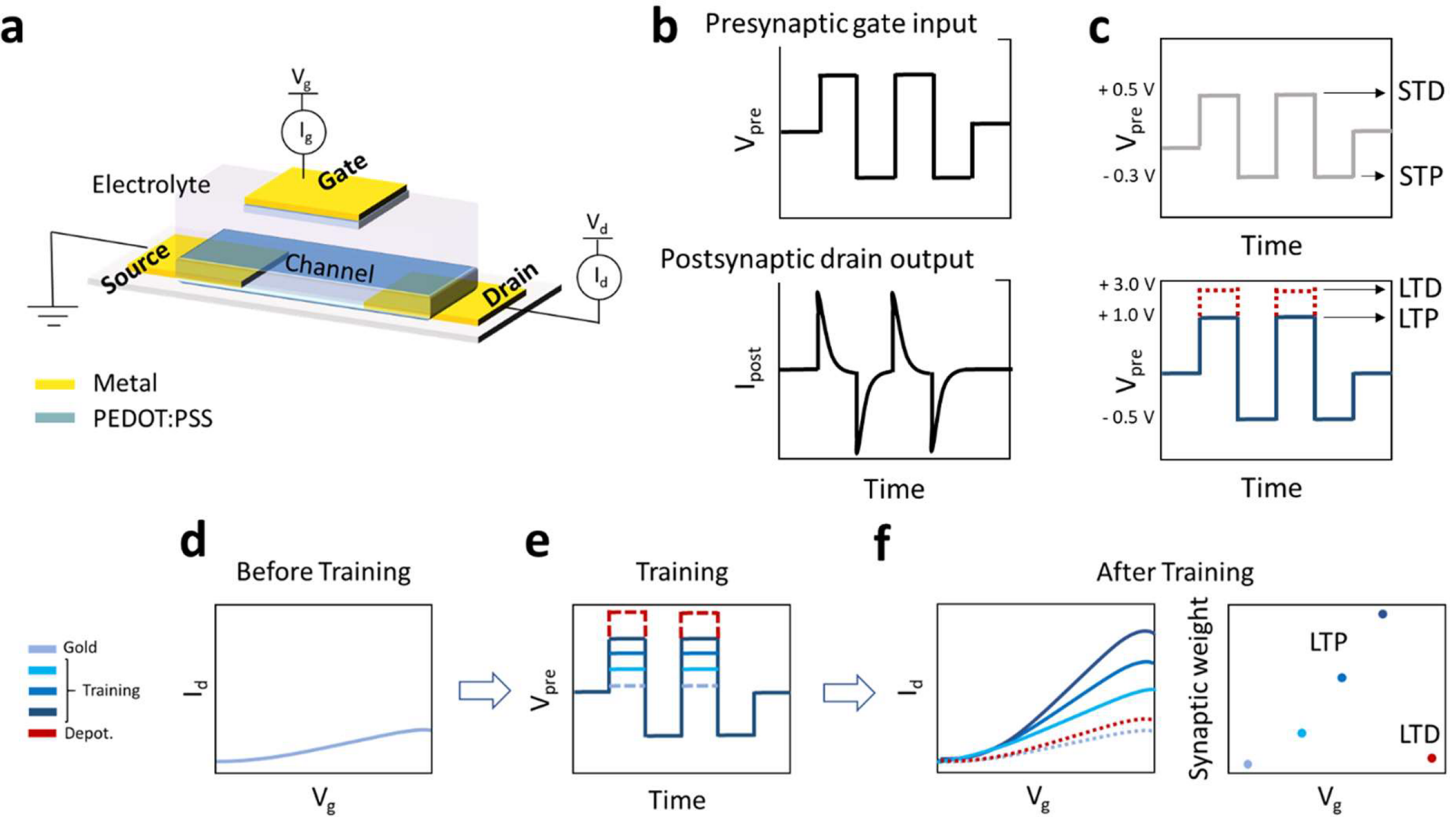
Operating principle of the neuromorphic OECT. (a) Sketch of OECT structure. (b) Sketch of presynaptic gate input and postsynaptic drain output. (c) Potential square waves employed for reading cycles and training cycles. (d–f) Effect of the training cycles (d) on transfer curve (f) for both LTP and LTD.

Long-term plasticity (LTP) has been studied with an approach that involves two steps: (i) a training step in which the plasticity is induced (Figure 1d); (ii) a reading step devoted to evaluate the extent of the enhancement (Figure 1c). The training phase involves the application of five stimuli consisting of five pulses each (pulse width 100 ms) with *V*_g_ varied from −0.5 to +1.3 V, while a *V*_d_ of −0.3 V is applied. After each training cycle, the plasticity effects on drain current (*I*_d_) were evaluated by varying *V*_g_ between −0.3 and +0.5 V (pulse width 100 ms) for a total of 160 pulses. The ability of *V*_g_ to modulate the conductivity of the channel was estimated by measuring the *I*_d_ variation realized during the reading steps and calculating the transconductance using the following formula:
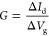
Finally, the ability of erasing the potentiation has been evaluated by applying 10 pulses (pulse width 100 ms) with *V*_g_ varied from −0.5 to 3 V, while a *V*_d_ of −0.3 V is applied. Also in this case, long-term depotentiation (LTD) was evaluated by a reading experiment performed as described above.

### Short-Term Plasticity

Short-term plasticity was evaluated by measuring *I*_d_ (*V*_d_ = −0.3 V) while five pulse pairs were applied to the gate in order to vary *V*_g_ from the baseline values of −0.5 to +0.5 V, with a pulse width of 1 ms and an interval between each pulse pair equal to 1 s. The experiment aims to evaluate the Δ*I*_d_ or Δ*G* (the two being equal, as Δ*V*_g_ is equal to 1 V) recorded between two pulses as a function of the time interval between them (Δ*t*), which was varied from 0.1 to 50 ms. The relaxation time constants (*t*_1_ and *t*_2_) of the neuromorphic device are calculated by fitting the Δ*I*_d_ (or Δ*G*) versus Δ*t* with a two-phase exponential decay function:
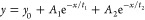
where *t*_1_, *t*_2_ and *A*_1_, *A*_2_ are the relaxation time constants and the amplitudes for rapid and slow decay, respectively.

### In Situ AFM Measurement

In situ atomic force microscopy (AFM) experiments were used to correlate the induced LTP and LTD with the morphological changes of the gate electrode topography. Measurements were performed in liquid using 0.1 mM PSS:Na and 10 mM EDOT solution. We performed three consecutive training procedures followed by a depotentiation protocol and a final training procedure. After each process, we measured the variation in the thickness of the gate electrode induced by the polymerization of PEDOT:PSS, and we acquired an image of the microstructure of the electrode surface. In parallel, we measured the electrochemical impedance spectroscopy (EIS) of the gate electrode versus the Ag/AgCl wire reference electrode. By fitting the obtained spectra with an equivalent circuit description, we were finally able to associate variation in the gate capacitance with changes in the electrode morphology induced by LTP and LTD.

## Results

### OECT Geometry and Working Principle

The OECT structure (Figures 1a and S1) is composed of a rectangular gold gate electrode (60 × 80 μm^2^) and a channel (60 × 260 μm^2^) made of PEDOT:PSS, with an average resistance of 1.18 ± 0.06 kΩ. The presynaptic signal (*V*_pre_) is the potential applied to the gate electrode, which acts as a controller of the drain current. Therefore, the drain current represents the postsynaptic signal (*I*_post_) (Figure 1b). The gate and the channel are immersed in a solution containing 0.1 mM Na:PSS and 10 mM EDOT. The experimental approach involves the use of training cycles followed by a device characterization, which is performed by applying *V*_pre_ values that do not induce LTP or LTD. Figure S2 shows the electronic and ionic circuit of the OECT and the processes occurring at the gate and channel for each experiment. The training cycles are carried out by applying *V*_pre_ pulses that are high enough to induce a structural modification of the device (Figures 1c and S2b,d). The *V*_pre_ application leads to the oxidation of the EDOT, which is followed by its polymerization on the surface of the gold gate electrode. Figure S2 shows the processes that occur during training cycles carried out to obtain LTP. On the other hand, LTD will be achieved by applying a sufficiently high potential to over oxidize PEDOT:PSS with a consequent loss of its electrical properties.

The effect of LTP and LTD was evaluated by characterizing the transistor by recording transfer curves and performing *V*_pre_ pulsed measurements, which do not lead to the polymerization of the PEDOT:PSS (Figure 1c). Figure 1d–f shows the effect of LTP and LTD on transfer curves. The native device exhibits a low *I*_post_ modulation (Figure 1d), since the gate electrode capacitance is only ascribable to the charge of the double electrical layer at gold-electrolyte interface (Figure S2). Following the application of the training cycles in the LTP mode (Figure 1e), a layer of PEDOT:PSS is deposited on the surface of the gate electrode. The transfer curves clearly show modification of the OECT structure and the effect of *V*_pre_ employed during LTP (Figure 1f). When the *V*_pre_ increases in a range wherein the polymerization occurs, the electropolymerization is boosted, and the slope of the transfer curve increases, highlighting the greater gate pseudocapacitance affecting the transistor operation. Conversely, once the training cycles are applied to induce LTD, a decrease in the slope of the transfer curves is observed, and it is attributed to PEDOT degradation causing a lower gate electrode capacitance. The behavior of the devices can be explained by considering the equations ruling the operation of the OECTs.

During LTP, the deposition of the PEDOT:PSS layer leads to an increase in the gate electrode capacitance, which permanently changes the operation of the transistor by increasing its ability to control the channel conductivity. In fact, the transconductance in the conditions used is equal to^24^
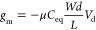
Where μ is the charge carrier mobility, *C*_eq_ the ionic circuit equivalent capacitance, *V*_d_ the drain-source potential, *W* the channel width, *d* the thickness, and *L* the length.

The value of *C*_eq_ is equal to
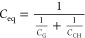
Where *C*_G_ is the ionic capacitance at the electrolyte-gate interface and C_CH_ is the channel capacitance.

Since PEDOT:PSS has a volumetric capacitance that affects the whole bulk of the material and not only its surface, its value is even 100 times higher than that of the double layer that is generated on a flat gold electrode,^25^ leading to an increase in the *C*_G_ value and consequent enhancement of *C*_eq_.

It is possible to explain how the amount of polymer deposited on the gate affects the operation of the device also through electrochemical processes **(**Figure S2**)**. In particular, in the native device, the application of positive *V*_pre_ leads to charging of the double electric layer of the gold gate electrode. The electrons will induce the following reactions at the channel:
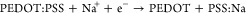
1

Once the training cycles that cause the electropolymerization of the PEDOT were carried out, the gate electrode was chemically modified. Since PEDOT:PSS exhibits a typical pseudocapacitive behavior, the application of the *V*_pre_ will induce redox reactions that are coupled with the ion diffusion into the materials leading to a significant increase of gate capacitance. In particular, the application of *V*_pre_ > 0 V will lead to the following reaction at the gate electrode:
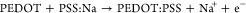
2

Since the number of electrons extracted depends on the amount of conductive polymer deposited, increasing the number of training cycles increases the entity of reaction 2 on the gate, with an effect on the functioning of the whole device. In fact, more electrons are available for reaction 1, leading to a larger gate action on the channel.

Conversely, the long-term depotentiation process is based on the polymer overoxidation, which leads to a loss of its electrical properties and therefore of its capacitance (Figure S2), depressing the gate actuation capability on the channel.

### AFM Investigation

**Figure 2:**
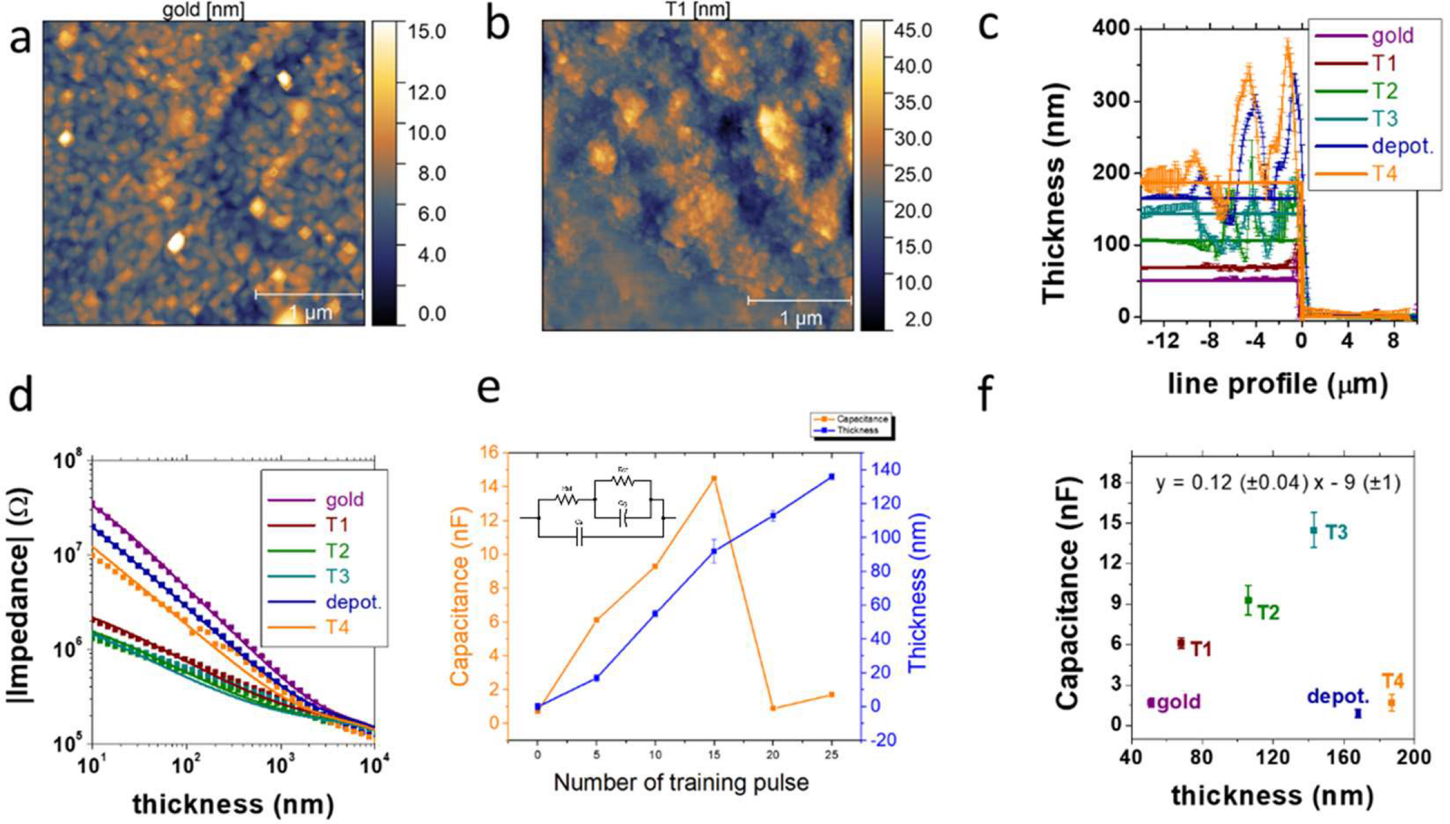
AFM images of the gate electrode surface before (a) and after (b) LTP. AFM profiles (c) and Bode plot (d) recorded at gate electrode for training cycles leading to LTP and LTD. (e) Trends of PEDOT:PSS thickness and capacitance values recorded during LTP and LTD (inset: equivalent circuit for capacitance estimation). (f) Correlation between gate capacitance and thickness during LTP.

The structural modifications generated during the training cycles were studied through atomic force microscopy (AFM) for clearly demonstrating PEDOT:PSS deposition at the gate electrode. Figure 2a shows AFM images of the gate electrode surface before and after LTP. In the image of the gold electrode, the grains of metal coating are visible, and the surface is characterized by a very low roughness value that is equal to 1.65 ± 0.04 nm. The gold film had a thickness of 51 ± 2 nm (Figure 2). This value was used as a reference point for the measurement of the electrodeposited PEDOT:PSS film thickness. The recording of the impedance spectrum, and the relative interpolation with a suitable equivalent circuit, allowed us to determine a *C*_eq_ equal to 0.7 ± 0.3 nF for the gold electrode. The application of a training cycle modifies the surface of the gate electrode as the typical grain structure of the PEDOT:PSS obtained by electrodeposition is clearly visible, and the material is homogeneously distributed over the entire electrode (Figure 2b). The PEDOT:PSS particles agglomerate in a more complex structures, and consequently, the roughness of the PEDOT:PSS film is equal to 4.7 ± 0.7 nm, a much higher value than that recorded for the gold surface. The effect of the deposition is also studied, measuring the thickness of the deposited PEDOT:PSS layer, which was 17 ± 3 nm. At the same time, the capacitance of the gate electrode increased up to a value of 6.1 ± 0.4 nF owing to PEDOT:PSS volumetric capacitance. Finally, the Δ*I*_post_ (Figure S3) values measured in the reading experiment are larger than that measured for the gold electrode, clearly highlighting the greater ability of the gate electrode to modulate the behavior of the channel. Repeating the training cycles (T2 and T3), an increase in both the PEDOT:PSS film thickness and electrode capacitance is observed (Figure 2c–e). The linear correlation between the gate electrode capacitance and the PEDOT:PSS film thickness demonstrates that all the conductive polymer is involved in the pseudocapacitive processes that occur on the material (Figure 2f). From the slope of the straight line, we determined the volumetric capacitance which is equal to (27 ± 7) F/cm^3^, whose value agrees with literature. The increased gate capacitance affects the operation of the neuromorphic device, leading to increased gate actuation capability on the channel, proved by the increasing Δ*I*_post_ in the reading phase after each LTP cycle. The roughness of the sample does not appear to be influenced by the successive cycles of LTP T2 and T3.

After doing three cycles of LTP (T1, T2, T3), we tested LTD. The LTD process leads to an increase in the thickness of the PEDOT:PSS film (from 143 ± 7 nm to 164 ± 4 nm) due to a residual polymerization of the material, which in any case occurs at the applied potential. However, the main process is the PEDOT:PSS overoxidation (depot), which destroys its conjugated structure by introducing oxygenated groups into the main chain and degrades its electrical properties. In fact, the impedance measurements show a clear decrease in the equivalent capacitance of the gate electrode, which decreases the Δ*I*_post_ measured in the reading phases. LTD leads to an increase in roughness together with slight morphological changes, presenting fewer but wider grains and structures on the PEDOT:PSS surface, which are both partially maintained after the following LTP.

After the LTD process, a further cycle of LTP (T4) was carried out, which led to an increase in the thickness of the polymeric film, with a slight increase in the value of gate capacitance demonstrating that the devices can undergo potentiation also after a depression process.

### Long-Term Plasticity: Effect of Gate Voltage

**Figure 3:**
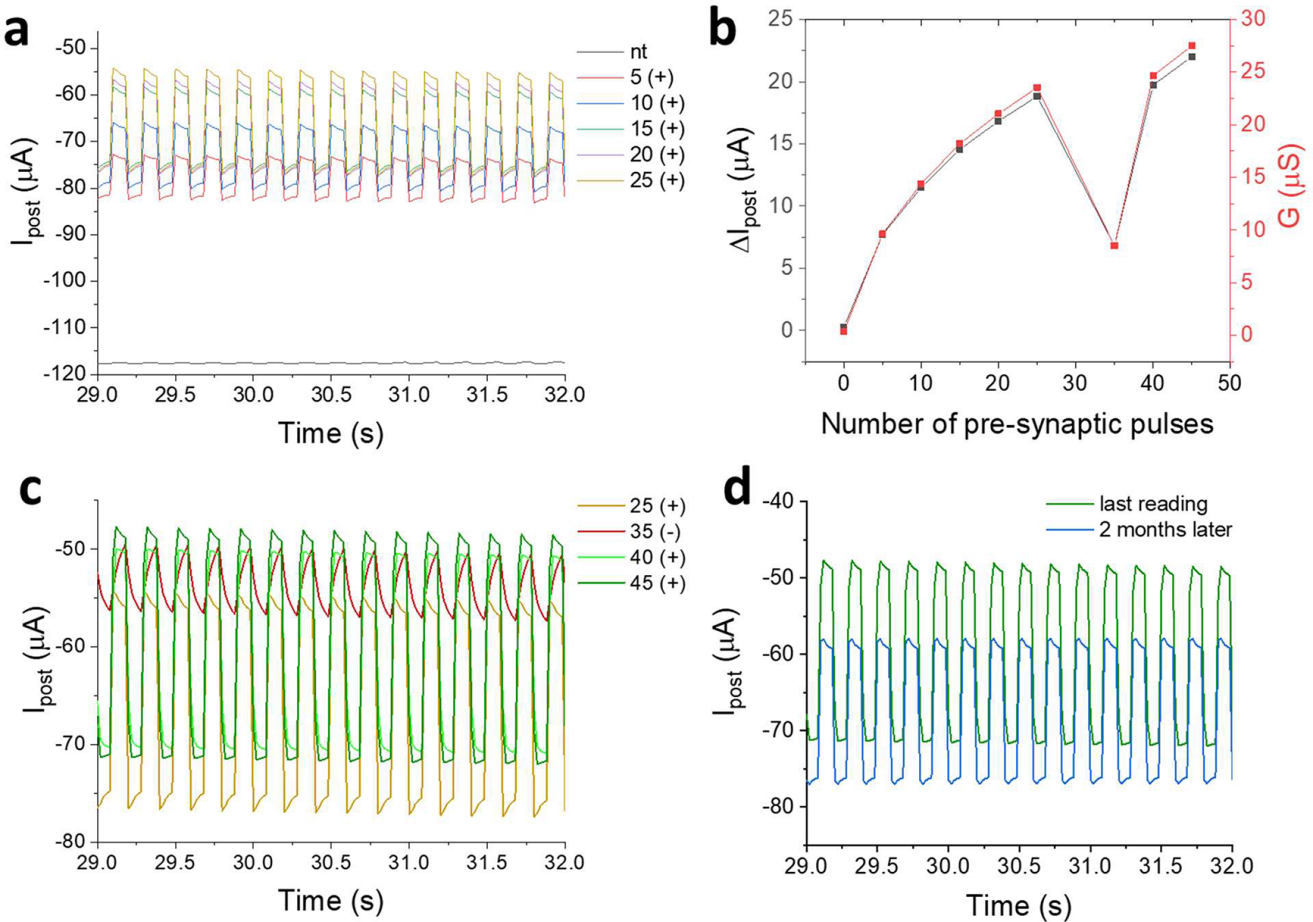
(a) *I*_post_ recording during reading experiments, using the native device and after 5, 10, 15, 20, and 25 cycles of training (*V*_pre_^MAX^ = +1.3 V). (b) Δ*I*_post_ value recorded versus the number of presynaptic pulses (*V*_pre_^MAX^ = +1.3 V). (c) *I*_post_ recording during reading experiments performed after 25 cycles of LTP (25+); 25 cycles of LTP (*V*_pre_^MAX^ = +1.3 V) followed by LTD (35−); LTD followed by 5 and 10 cycles of LTP training (40+ and 45+) (*V*_pre_^MAX^ = +1.3 V). (d) *I*_post_ recording during reading experiments immediately after LTP and after 2 months.

The effect of the number of LTP impulses was studied by applying five stimuli to the same potential, comprising five pulses each. The detailed experimental procedure is reported in Table S1, and the results are shown in Figures S4–10. Figure S9d shows the shape of the curve of *V*_pre_, in which the maximum potential value was equal to +1.3 V. *V*_pre_^MAX^ was varied in order to study the effect of the polymerization voltage on the device response. Figure S11 shows a typical trend of gate current (*I*_g_) as a function of time during a stimulus cycle. It is possible to observe that following each positive potential pulse, there is a strong *I*_g_ increase connected to the PEDOT electropolymerization process in accordance with the polymerization reaction:
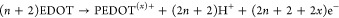
Where (*n* + 2) is the number of monomers that form the polymeric chain, while *x* is the number of holes in the conductive polymer. Following each training, the reading test is carried out. The reading test (potential curve in Figure S9f) was studied by applying *V*_pre_ pulses (−0.3 V < *V*_pre_ < + 0.5 V), while *I*_post_ generated by a drain voltage (*V*_d_) equal to −0.3 V was measured. The difference (Δ*I*_post_) between the *I*_post_ values measured at *V*_pre_ equal to 0.5 V and −0.3 V provides a useful indication of the *V*_pre_ ability to modulate the conductivity of the channel. The higher the average Δ*I*_pre_, calculated as the arithmetic mean of five Δ*I*_post_ variations, the larger the modulation obtained in the OECT channel and therefore the better the result of the long-term enhancement will be. Typically, a reading experiment was performed following each stimulus, and it is therefore possible to obtain the trend of Δ*I*_post_ as a function of the number of stimuli. Figure 3b shows the reading curves recorded for the native device and following 5, 10, 15, 20, and 25 training pulses. The Δ*I*_post_ grows as the number of stimuli increases, starting from an almost zero value recorded for the nonenhanced device (Figure 3d). This result highlights an enhancement of the gate’s ability to modulate *I*_post_, demonstrating the ability of the device to effectively emulate long-term plasticity.

After the last train of LTP pulses, the possibility of obtaining a LTD was usually also verified, by applying a *V*_pre_^MAX^ = 3 V (Figure S9e), capable of degrading the polymer deposited on the gate by overoxidation reactions. Furthermore, the possibility of manifesting LTP after LTD was verified by applying additional training cycles. Figure 3c shows the reading curves after the LTD and after the subsequent cycles of LTP. As expected, the application of a series of pulses at 3.0 V leads to a decrease in the Δ*I*_post_ in reading experiments, highlighting a decrease in the gate actuation capacitance. Following subsequent training pulses such as to cause the deposition of the PEDOT:PSS, the increase of the gate’s ability to modulate the *I*_post_ is observed, highlighting the successful repotentiation.

It is important to note that the changes brought about by the training cycles are structural and last for a long time. As can be seen in Figure 3e, the *I*_post_ variation in the reading cycles remains almost constant even after 2 months from the enhancement cycles, highlighting the good performance of this approach. The variations in the performance of the device can also be estimated in terms of the variation of the mean transconductance calculated as the ratio between Δ*I*_post_ and the Δ*V*_pre_.

**Figure 4:**
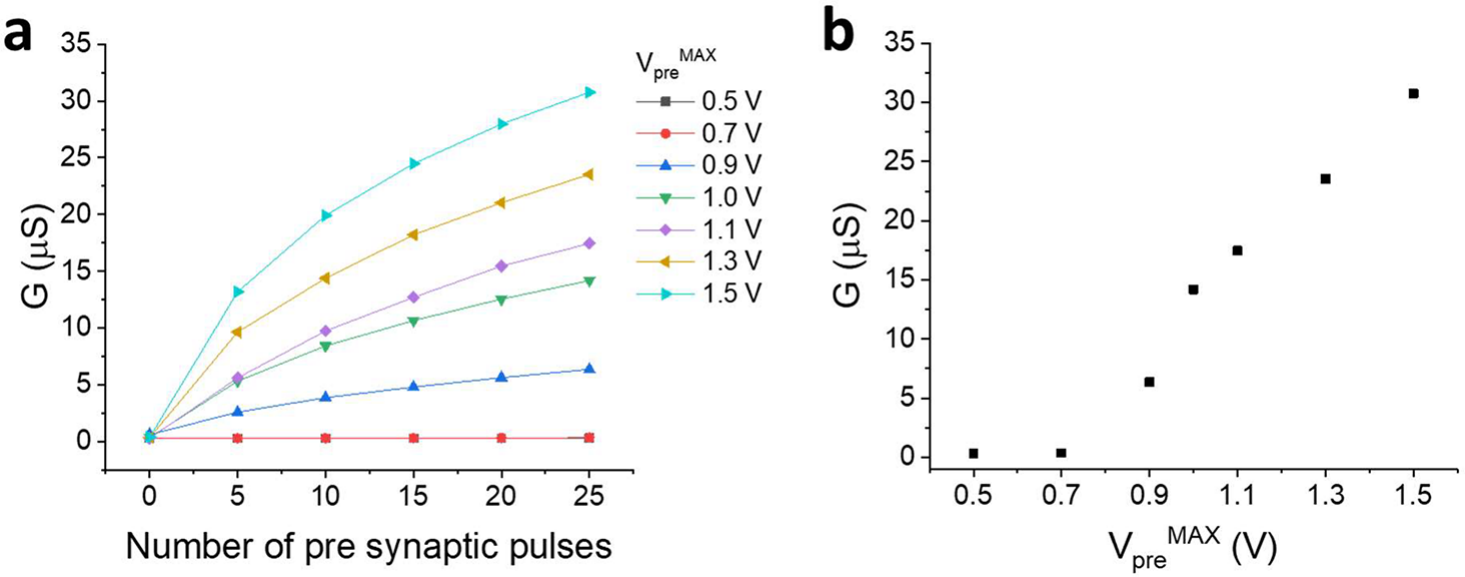
(a) Transconductance recorded after LTP cycles performed with pulses at different *V*_pre_ values. (b) Transconductance recorded after 25 pulses of training vs the *V*_pre_^MAX^ used for the pulse.

Furthermore, the effect of *V*_pre_^MAX^ applied during the training was evaluated in terms of transconductance. Figure 4a shows the trend of the transconductance measured in the reading phase, when the *V*_pre_^MAX^ applied during the training phase was varied between 0.5 and 1.5 V (rough data are reported in Figures S12 and S4–10). The reported results show that the *V*_pre_^MAX^ plays a key role in generating the neuromorphic behavior of the device, and Figure 4b shows the trend of the transconductance measured at the 25th cycle as a function of the *V*_pre_^MAX^ applied during training. It is possible to observe that no variation of the transconductance is observed following training experiments wherein the highest *V*_pre_^MAX^ is lower than or equal to 0.7 V, because the applied voltage is not able to induce the polymerization of the PEDOT:PSS on the gate electrode. Similarly, *V*_pre_^MAX^ values higher than 0.7 V lead to a neuromorphic effect and the observed transconductance increase by increasing to the applied *V*_pre_^MAX^. The results obtained suggest the presence of a threshold potential, above which a long-term enhancement in the neuromorphic device can be observed. Although this phenomenon occurs in biological system in a more complex way and is dependent on many factors, neurons exhibit thresholds as well above which the phenomena of LTP and LTD are manifested.^26^

### Short-Term Plasticity

**Figure 5:**
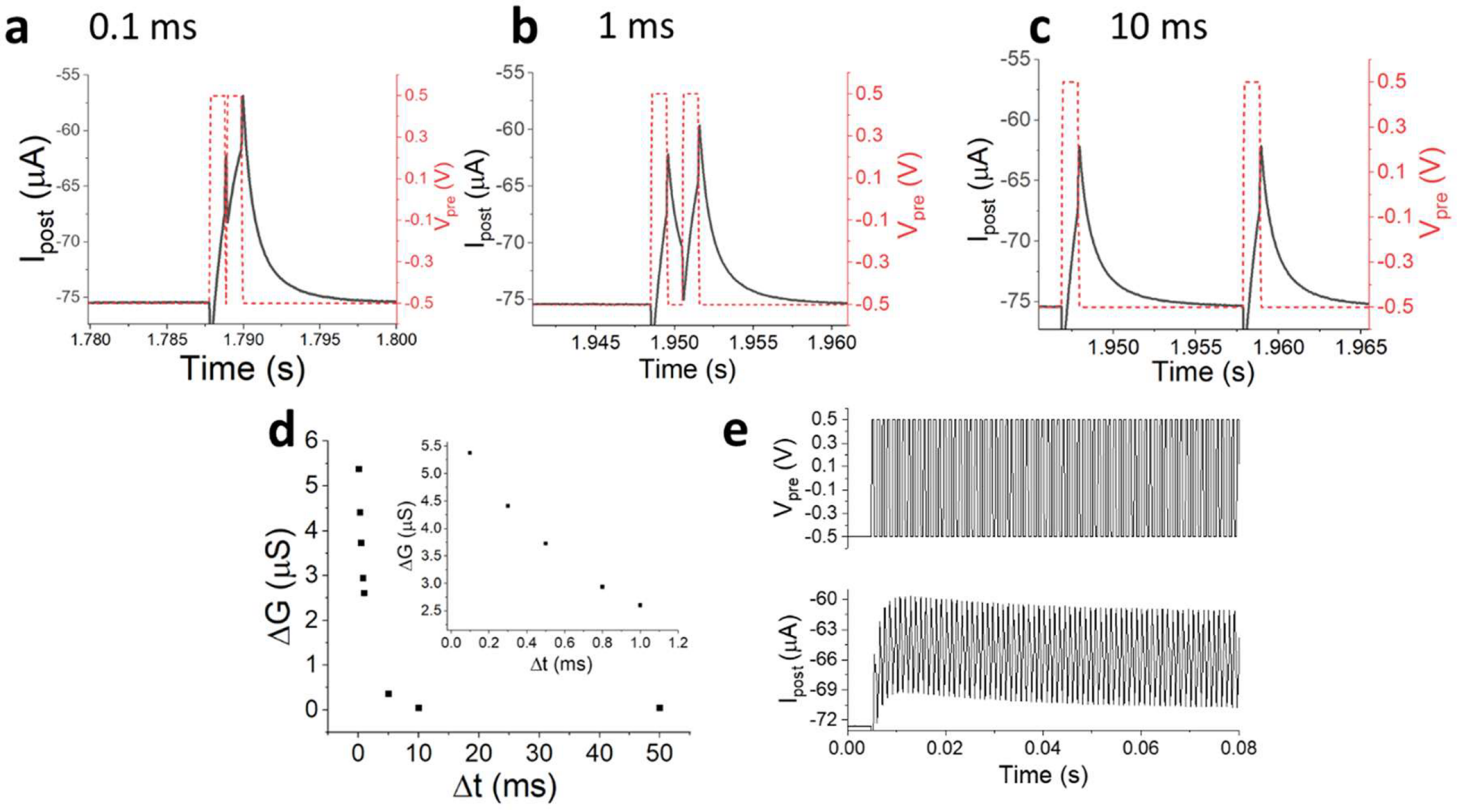
*I*_post_ recorded upon application of pairs of *V*_pre_ pulses (pulse width 1 ms) with Δ*t* of 0.1 (a), 1 (b), and 10 (c) ms between the pulses for a device trained with *V*_pre_^MAX^ = 1.3 V. (d) Transconductance variation measured between the two *I*_post_ peaks recorded during the application of the *V*_pre_ pulses to stimulate paired pulse depression (PPD) vs Δ*t* (inset: zoomed-in from 0.1 to 1 ms). (e) Plastic adaptation to PPD when a train of *V*_g_ pulses (Δ*t* = 1 ms) is applied.

Short-term plasticity was investigated by measuring the *I*_post_ during the application of two closely spaced *V*_pre_ pulses (Δ*t* < 100 ms). Ideally, the measured values should depend only on the *V*_pre_; however, neuromorphic devices exhibit hysteresis effects, which lead to a difference (Δ*I*_post_) between the *I*_post_ values measured during the first and second pulses. Figure 5a shows a typical curve of *I*_post_ as a function of time, during the application of two *V*_pre_ pulses with a Δ*t* of 0.1 ms. The *I*_post_ measured following the second pulse turns out to be lower in absolute value than that measured during the first pulse, thus confirming the short-term plasticity for the studied device and, in particular, the occurrence of a paired pulse depotentiation (PPD). By increasing the time between the two pulses, the effect decreases as shown in Figure 5b (Δ*t* = 1 ms), until no effect is observed for Δ*t* equal to 10 ms (Figure 5c). Also in this case, it is possible to analyze the response in terms of transconductance variation (Δ*G*) measured between the first and second pulse. Figure 5d shows the trend of Δ*G* as a function of the time between the two pulses. The reported data can be interpolated with a two-phase exponential decay function (Figure S13), as described in the Experimental Section. Among the fitting parameters, t1 and t2 represent the time constants for the rapid and slow decay phases, respectively. For the device used in the experiments showed in Figure 5, i.e., trained with *V*_pre_^MAX^ equal to 1.3 V, we obtain 0.4 and 2.0 ms. These results are in good agreement with the ionic time constant calculated from the equivalent circuit of the OECT, i.e., 2.5 ms. The effect of *V*_pre_^MAX^ on t1 and t2 was investigated for OECTs trained with *V*_pre_^MAX^ equal to 0.9, 1.1, and 1.3 V and without potentiation (Figure S14). For the latter, no STP occurs due to the small capacitance of the bare Au gate electrode (as expected when calculating the OECT ionic time constant, which is 0.14 ms), but for all devices trained using *V*_pre_^MAX^ above the threshold, STP is observed. However, no clear correlation with the time constant values can be established as the high speed of the transistors likely dominates over small *C*_G_ variations among the differently trained devices. Figure 5e shows the plastic adaptation of the device to PPD, when a train of pulses is applied with Δ*t* = 1 ms. After the fifth pulse, no PPD was observed anymore.

## Discussion

OECTs are emerging devices used as artificial synapses because they can emulate several neurological processes. However, LTP phenomena are still difficult to simulate with an OECT due to a rapid loss of the redox state of the conducting polymer, which constitutes the device channel. The modification of the transistor structure through electropolymerization processes has shown very promising results, characterized by a long maintenance of the induced neuromorphic state.

The approach used in this work involves the polymerization of PEDOT:PSS on the gate electrode, in order to modify its electrical capacity and therefore its ability to actuate on the channel. On one hand, the action of the presynaptic stimulus acts as an action potential in signal transmission by inducing the postsynaptic signal. On the other hand, AFM investigations clearly show that the action potential induces a modification of the structure of the gate electrode and therefore generates the LTP behavior of the neuromorphic device. The weight of the synapse can be estimated by measuring the transconductance, which, in the experiments carried out, varies from 0.3 μS for the native device to 30 μS for the neuromorphic OECT with the highest LTP and depends both on the strength of the presynaptic signal and on the number of used pulses. LTP is observed for action potentials exceeding the electropolymerization threshold of EDOT (*V*_g_ > 0.7 V), in analogy to what has already been observed for neurons for which LTP is induced at action potentials exceeding a specific biological threshold.^26^ From the energy consumption evaluated in the training phase, the artificial synapse presents an energy absorption of about 30 nJ for each potential pulse necessary for the deposition of the PEDOT:PSS. Although the energy consumption is much higher than 1 pJ, which is the highest value observed for a biological synapse,^6^ these results are very close to the one of a neuromorphic device based on the polymerization of PEDOT:PSS dendrites in the channel.^16^

Although no practical trials have been performed, the dual role of the presynaptic signal as an action potential and as a stimulus of the LTP suggests that this artificial synapse can be trained directly during operation, adapting to changing environmental conditions. The structural strengthening of the artificial synapse is stable for at least two months, and the behavior can be reset by inducing LTD and therefore potentiated again. It is worthy to note that LTP appears to be reversible from a phenomenological point of view, as it is possible to enhance the device again through LTP cycles after performing the LTD. However, the overoxidation leads to the formation of a nonconductive layer on the gate electrode, and therefore, the process could be not reversible from a structural point of view, with related consequences on the neuromorphic behavior of the OECT. The formation of an insulating layer during LTD could generate a potential drop, which could hinder the achievement of a good reversibility in LTP/LTP cycles.

Our brain undergoes not only long-term modifications but also temporary changes and adaptive dynamics typical of short-term plasticity, which is essential for decoding temporal information in biological systems.^27^ The artificial synapse also mimics short-term plasticity, and in particular paired pulse depression, with two distinguishable decay phases in analogy with some biological synapses^28^ and other neuromorphic devices.^29^ Nevertheless, the time constants associated with STP in our device, i.e., 0.4 and 2.0 ms, are considerably smaller than those characterizing the decay phases reported in some biological synapses, where the fast and slow decays range from tens to hundreds of ms or more^28,30^ and make this neuromorphic transistor one of the fastest among other state-of-the-art artificial synapses based on OECTs.^10,29,31,32^ It is worth noting that PPD and LTP were induced using different shapes of *V*_pre_ waves with the same experimental setup. These results suggest that the proposed device could combine short-term plasticity and long-term plasticity in a hybrid process. Moreover, the LTP also affects the STP. No STP was measured for pristine OECTs endowed with a gold electrode (Figure S14), while it was observed for devices that had been trained (Figure 5). The STP threshold time is correlated to the OECT time constant. In order to provide a memory effect, consecutive pulses need time delays shorter than the time constant, so as to have the equivalent capacitance partially charged once the second pulse starts. The shorter the Δ*t* between the first two pulses, the more pronounced the resulting Δ*G*, since the capacitance has less time for discharging. This explanation is confirmed by the decrease of the Δ*t* threshold value for OECTs having a bare gold gate electrode for OECTs, because the expected equivalent capacitance leads to an OECT time constant of 0.14 ms. The role of gate and channel capacitances in LTP and STP is thoroughly discussed in the SI.

The performance of our device has been compared with the performance of other electrochemical transistors used for neuromorphic applications (Table 1). The approach initially involved the use of the gate electrode as a writing element and the neuromorphic behavior was accomplished through the modification of the redox state of the channel polymer. Usually, the authors claim only two states of writing, while the retention of the state appears to be between a few seconds and a few hours depending on the different employed materials. In order to increase the retention times, devices have been developed by inducing an irreversible structural modification that stabilizes the redox state of the polymer. In particular, the structural collapse due to PEDOT:PSS reduction was exploited by Winther-Jensen et al. for the realization of a neuromorphic device able to maintain the conductive state for at least 20 h. Salleo’s group systematically investigated the use of channels made of PEDOT:PSS blended with PEI (polyethylenimine).^10,38^ During reduction of PEDOT, the PEI ammine groups are protonating, and so, PEI screens the conductive polymer backbone from the PSS negative charge. Also, thanks to the encapsulation of the device, it was possible to obtain a retention of more than 25 h. In order to increase the retention times of the neuromorphic modification, devices based on the electropolymerization of a conductive polymer between two gold electrodes have been developed, so as to obtain a conductive trace which modifies the functioning of the device. The structural modification of the device allows to overperform the previous approach maintaining the induced neuromorphic state for a few months. Similarly to devices based on electropolymerization, the device described here allows to maintain the neuromorphic state for 2 months, with a continuous distribution of states. Since the other neuromorphic properties presented for the OECTs have also been demonstrated for our device, we believe the approach used is very promising.

**Table 1:** Performance of Neuromorphic Devices That Exhibit LTP

Channel material**/**electrolyte**/**gate material	Number of states	Memory mechanism	State retetion/demostrated cycles	ref.
poly(3-methylthiophene)/poly(ethylene oxide-propylene oxide) + LiCIO_4_/–	4	counter-redox reaction	hours/–	(13)
poly[2-methoxy-5-(2′-ethylhexyloxy)-p-phenylenevinylene]/RbAg_4_I_5_/AlTi	8	diffusion disparity	>240 h/–	(33)
poly(3,3‴-didodecylquaterthiophene/poly(ethylene oxide) + LiClO_4_ + ethyl viologen/Au	2	counter-redox reaction	14 h^34^/>100^35^	(34, 35)
polyaniline/poly(ethylene oxide) + LiClO_4_/silver	>2	slow kinetics	–/104	(36)
PEDOT:poly(tetrahydrofuran)/KCl/Ag/AgCl	continuous	slow kinetics + structural rearrangement of the polymer	<1 s/–	(37)
PEDOT:poly(tetrahydrofuran)/NaCl/Ag/AgCl	2	structural collapse during electrochemical reduction	>20 h/>50	(15)
PEDOT:PSS/poly(vinylidenefluoride-*co*-trifluoroethylene)/conductive carbon	2	ferroelectric polarization	hours/–	(14)
polysodium 4-(2-(2,5-bis(2,3-dihydrothieno[3,4-*b*][1,4]dioxin-5-yl)thiophen-3-yl)ethoxy)butane-1-sulfonate/NaCl/Ag/AgCl	continuous	electropolymerization in the channel	months/–	(17)
dendritic PEDOT:PSS/NaPSS + EDOT + benzoquinone + hydroquinone/Ag/AgCl	continuous	electropolymerization in the channel	not specified	(16)
PEDOT:PSS + poly(ethylenimine)/KCl/PEDOT:PSS	>500	variation of redox state	25 h/–	(10)
PEDOT:PSS + poly(ethylenimine)/KCl/PEDOT:PSS	continuous	variation of redox state	680 s/–	(38)
PEDOT:PSS/EDOT + NaPSS/PEDOT:PSS	continuous	variation of gate capacitance	2 months/–	our device

## Conclusion

This work explores the neuromorphic behavior evoked by the deposition of PEDOT:PSS on the gate electrode of an OECT, used to induce long-term plasticity persisting for at least two months. Innovatively, the neuromorphic effect does not use the channel as a memory element but affects the ability of the transistor to work as a controller. The device operation was demonstrated by monitoring the PEDOT:PSS deposition on the gate electrode through operando AFM measurements and EIS characterization, providing new insights on the widely used electropolymerization processes. Similarly to the neuron behavior, the device shows a threshold potential that must be exceeded to induce long-term potentiation. Since the gate voltage has the double function of action potential and stimulus necessary for long-term potentiation and depotentiation, this device will be able to adapt its behavior to changing environmental conditions during real use. This approach can be further improved by using different materials and optimizing the device architecture, and the integration in neuromorphic circuits opens fascinating perspectives in the realization of advanced neuromorphic circuits based on OECTs. Finally, long-term potentiation is based on consolidated electrochemical processes generating a gate modification that is easily described by the OECT quantitative model. The deep knowledge of the operation of the OECT will allow a smart design of the device to reach the technical specifications required for neuromorphic computing applications.

## Supporting Information Available

OECT architecture, chemical processes occurring during LTP and LTD, *I*_post_ vs time curve recorded during AFM experiment, experimental results obtained for training pulses having different *V*_pre_^MAX^, *I*_g_ vs time curve recorded during training cycle, *I*_post_ vs time recorded after 25 pulses of training at different *V*_pre_^MAX^, exponential fit of paired pulse depotentiation experiments for OECTs trained at different *V*_pre_^MAX^, detailed procedure of the training scheme (PDF)
