# Supplementary material for: High-Endurance Long-Term Potentiation in Neuromorphic Organic Electrochemical Transistors by PEDOT:PSS Electrochemical Polymerization on the Gate Electrode
Source: ACS Appl Mater Interfaces. 2023 Nov 15;16(45):61446–56. doi: 10.1021/acsami.3c10576 (PMC11565569; doi:10.1021/acsami.3c10576)
Supplement: Supplementary file 1 — am3c10576_si_001.pdf [file am3c10576_si_001.pdf]

Supporting information

High endurance long term potentiation in neuromorphic organic electrochemical transistors by PEDOT:PSS electrochemical polymerization on the gate electrode

Federica Mariani[1], Francesco Decataldo[2], Filippo Bonafè[2], Marta Tessarolo[2], Tobias Cramer[2], Isacco Gualandi[1]*, Beatrice Fraboni[2], Erika Scavetta[1]

[1] Department of Industrial Chemistry "Toso Montanari", Alma Mater Studiorum - University of Bologna, Viale del Risorgimento 4, 40136 Bologna, Italy

[2] Department of Physics and Astronomy, Alma Mater Studiorum - University of Bologna,, Viale Berti Pichat 6/2, 40127 Bologna, Italy

Corresponding Author. Isacco Gualandi, Department of Industrial Chemistry "Toso Montanari", Alma Mater Studiorum - University of Bologna, Viale del Risorgimento 4, 40136 Bologna, Italy, +390512093386, isacco.gualandi2@unibo.it

List of the contents

Figure S1. OECT architecture

Figure S2. Chemical processes occurring during LTP and LTD

Figure S3. I_{post} vs time curve recorded during AFM experiment

Figure S4. LTP experiments with V_{pre}^{MAX} = +0.5 V

Figure S5. LTP experiments with V_{pre}^{MAX} = +0.7 V

Figure S6. LTP experiments with V_{pre}^{MAX} = +0.9 V

Figure S7. LTP experiments e with V_{pre}^{MAX} = +1.0 V

Figure S8. LTP experiments with V_{pre}^{MAX} = +1.1 V

Figure S9. LTP experiments with V_{pre}^{MAX} = +1.3 V

Figure S10. LTP experiments with V_{pre}^{MAX} = +1.5 V

Figure S11. I_g vs time curve recorded during training cycle for V_{pre}^{MAX} equal to + 1.3 V

Figure S12. I_{post} vs time recorded after 25 pulses of training at different V_{pre}^{MAX}

Figure S13. Exponential fit of paired pulse depotentiation experiments.

Figure S14. Exponential fit of paired pulse depotentiation experiments for OECT trained at different V_{pre}^{MAX}

Table S1. Detailed procedure of the training scheme

Additional details SI 1 Roles of gate and channel capacitances in LTP and STP

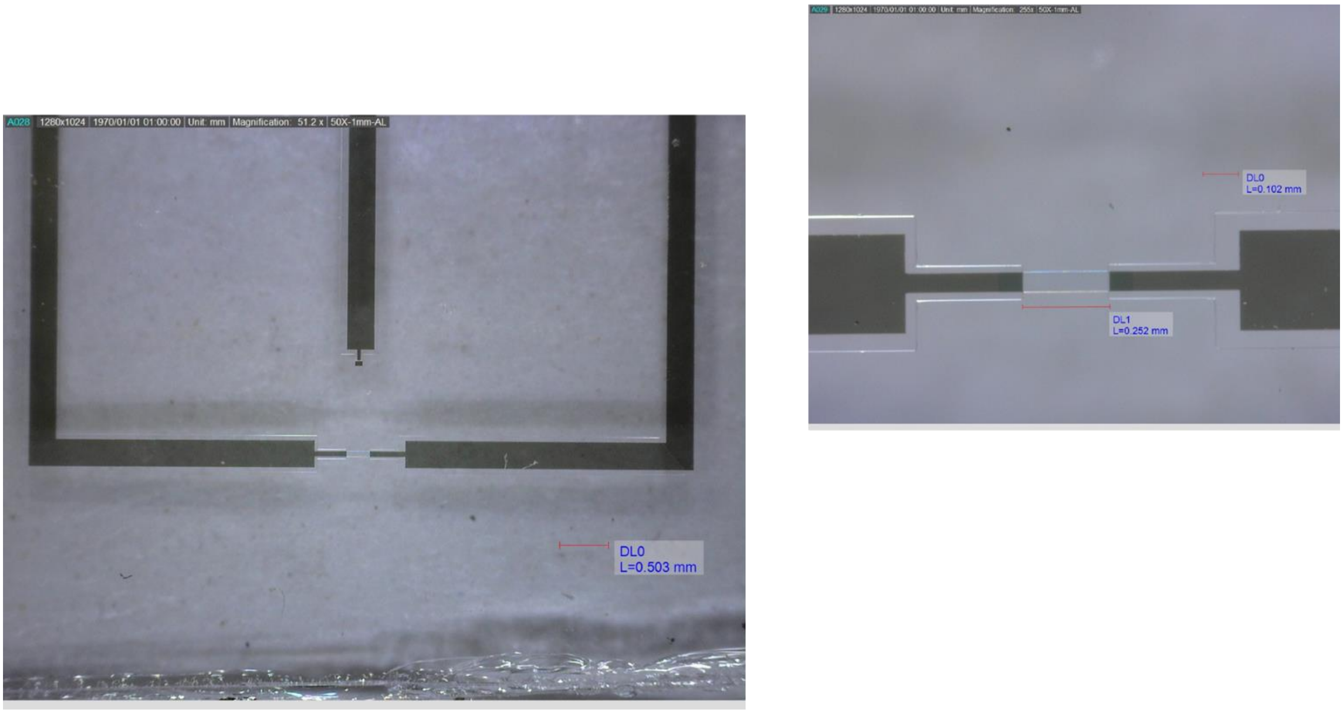

Figure S1. OECT architecture

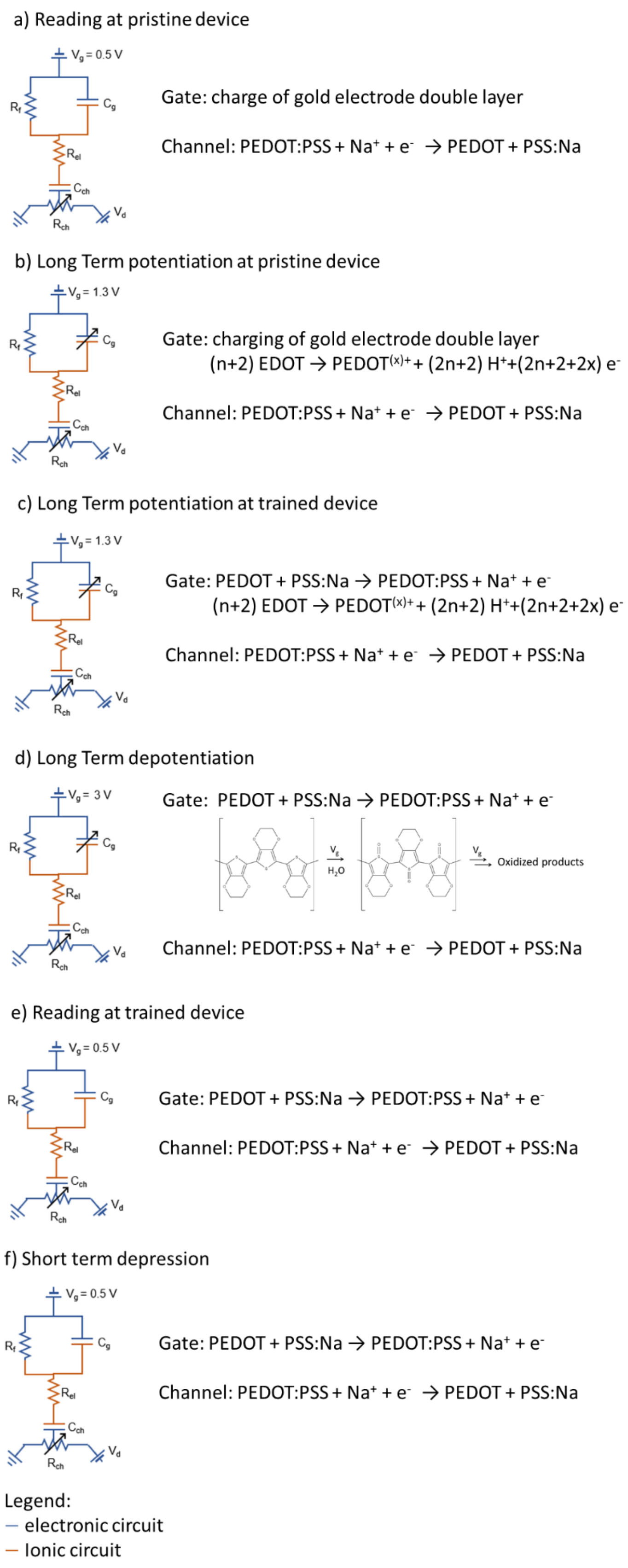

Figure S2 Chemical processes occurring during experimental processes: a) reading at pristine devices; b) LTP at pristine device; c) reading at trained device; d) LTP at trained device; e) LTD; f) short term depression

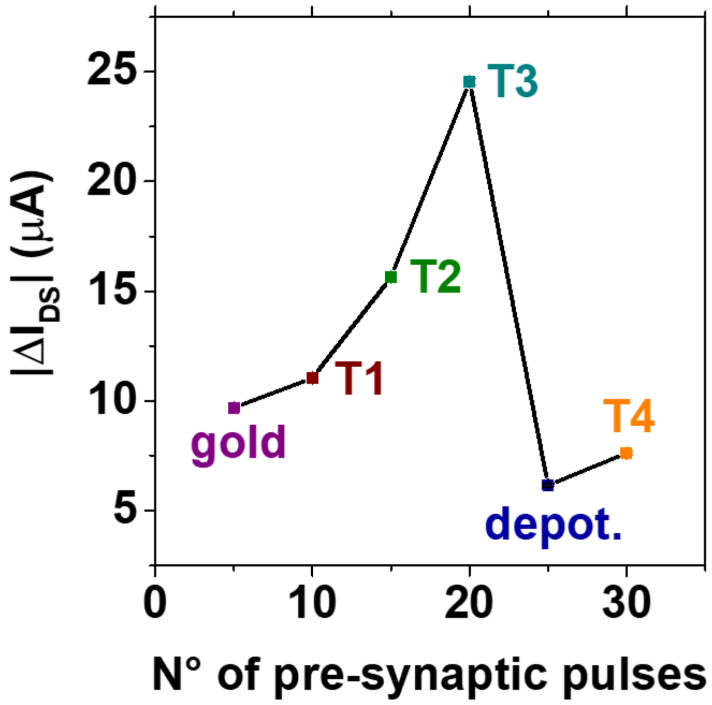

Figure S3 I_{post} vs time curve recorded during AFM experiment

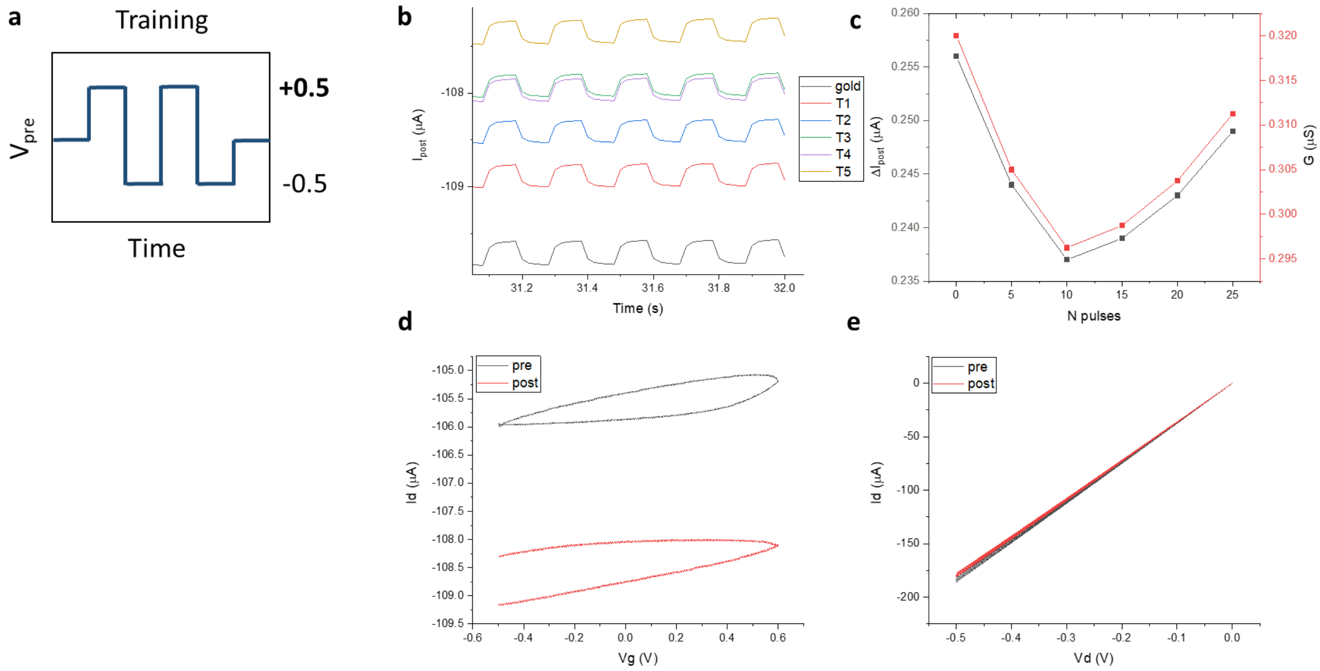

Figure S4 Voltage wave used during training experiments (V_{pre}^{MAX} = +0.5 V) (a), I_{post} vs time recorded in reading experiments (b), ΔI_{post} vs number of pulses (c), transfer curve before and after training experiments (d) and output curve recorded before and after training experiment (e).

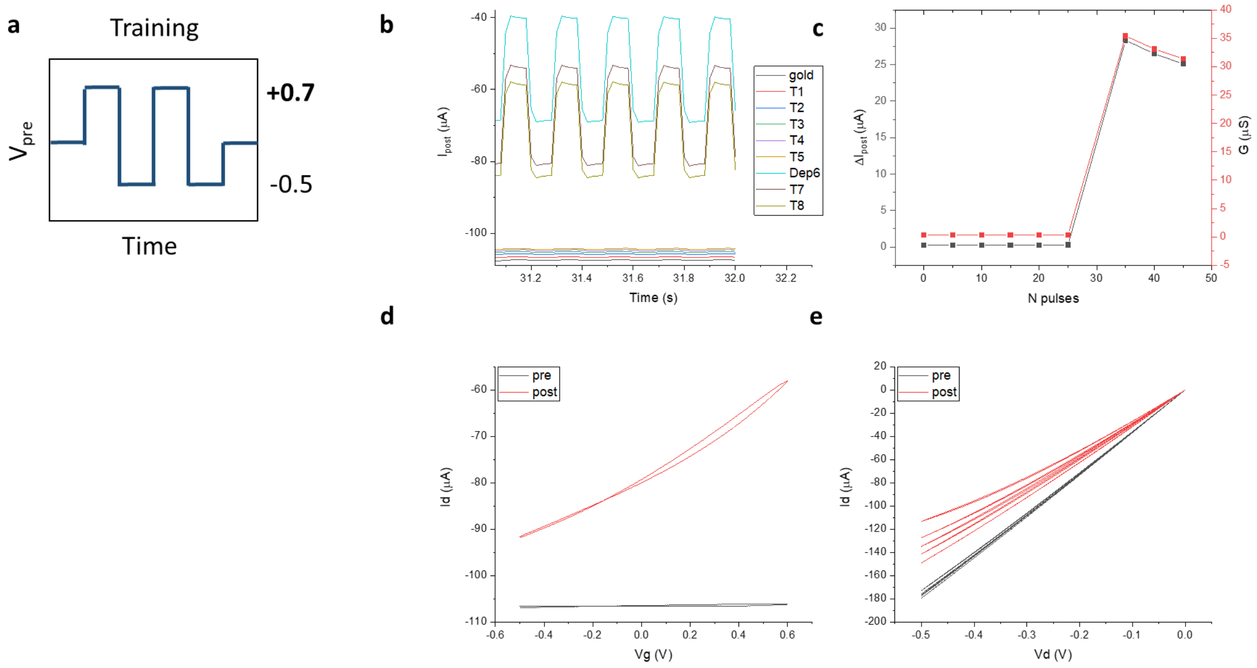

Figure S5 Voltage wave used during training experiments (V_{pre}^{MAX} = +0.7 V) (a), I_{post} vs time recorded in reading experiments (b), ΔI_{post} vs number of pulses (c), transfer curve before and after training experiments (d) and output curve recorded before and after training experiment (e).

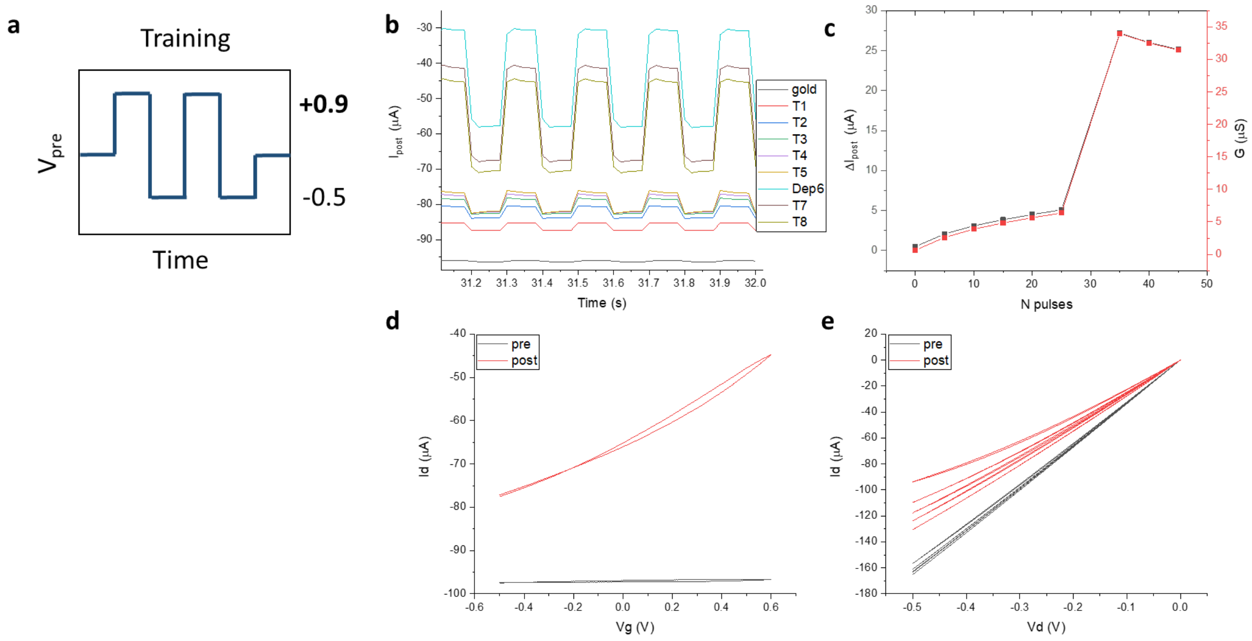

Figure S6 Voltage wave used during training experiments (V_{pre}^{MAX} = +0.9 V) (a), I_{post} vs time recorded in reading experiments (b), ΔI_{post} vs number of pulses (c), transfer curve before and after training experiments (d) and output curve recorded before and after training experiment (e).

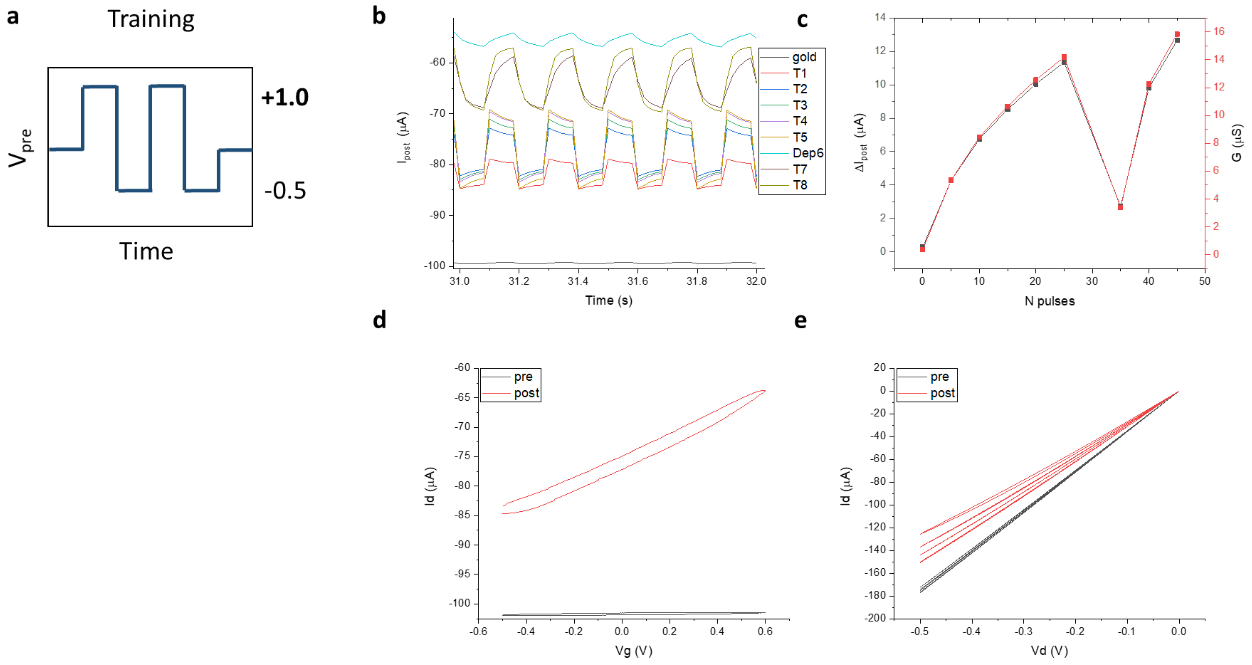

Figure S7 Voltage wave used during training experiments (V_{pre}^{MAX} = +1.0 V) (a), I_{post} vs time recorded in reading experiments (b), ΔI_{post} vs number of pulses (c), transfer curve before and after training experiments (d) and output curve recorded before and after training experiment (e).

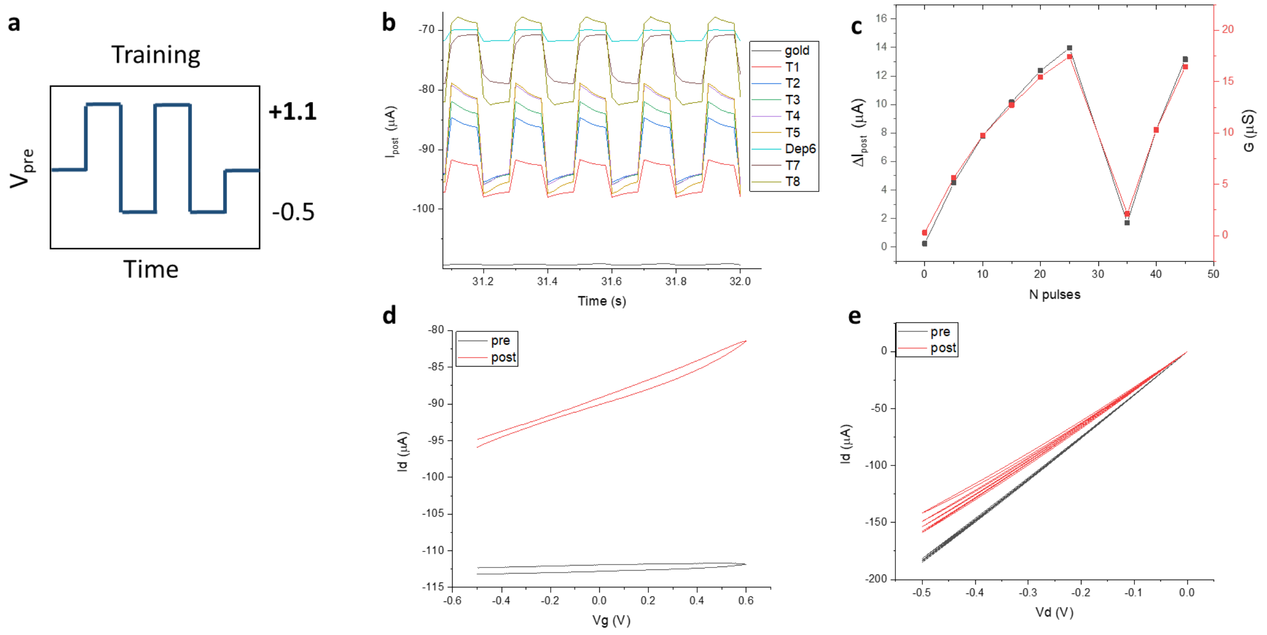

Figure S8 Voltage wave used during training experiments ($V_{pre}{}^{MAX}$ = +1.1 V) (a), I_{post} vs time recorded in reading experiments (b), ΔI_{post} vs number of pulses (c), transfer curve before and after training experiments (d) and output curve recorded before and after training experiment (e).

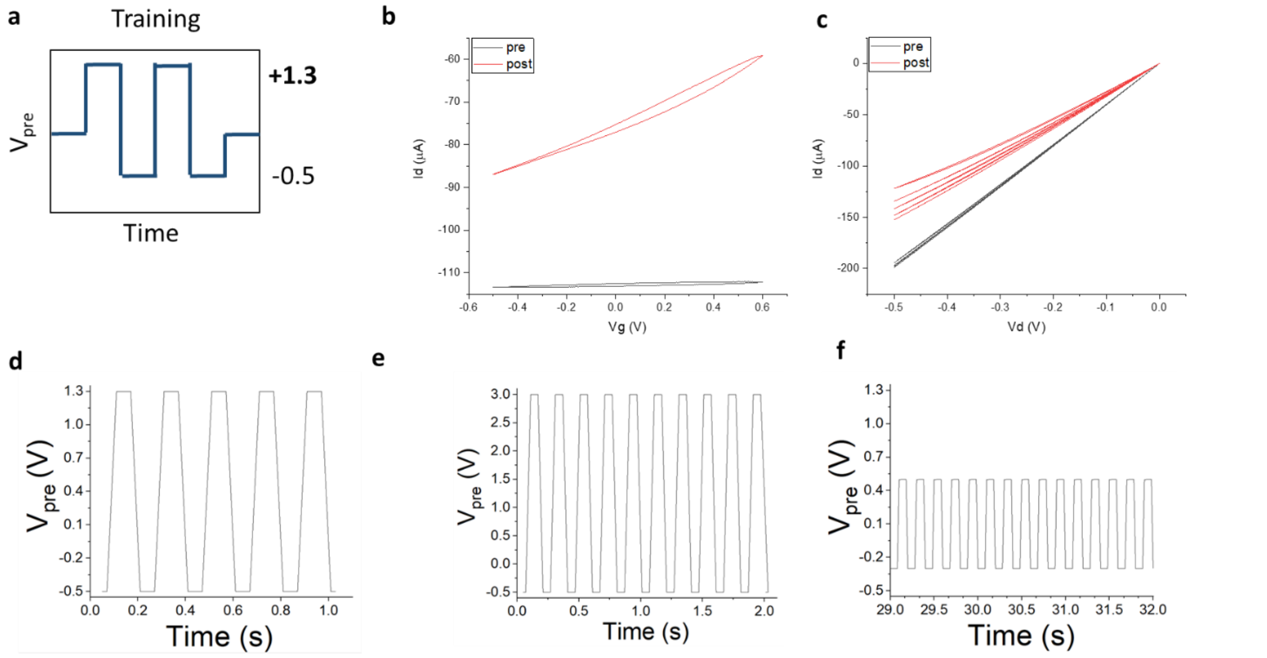

Figure S9 Voltage wave used during training experiments ($V_{pre\ max}$ = +1.3 V) (a and d), transfer (b) and output (c) curves recorded before and after training experiments. Voltage waves used during training to obtain LTP (d) and LTD (e) and during all reading (f) experiments.

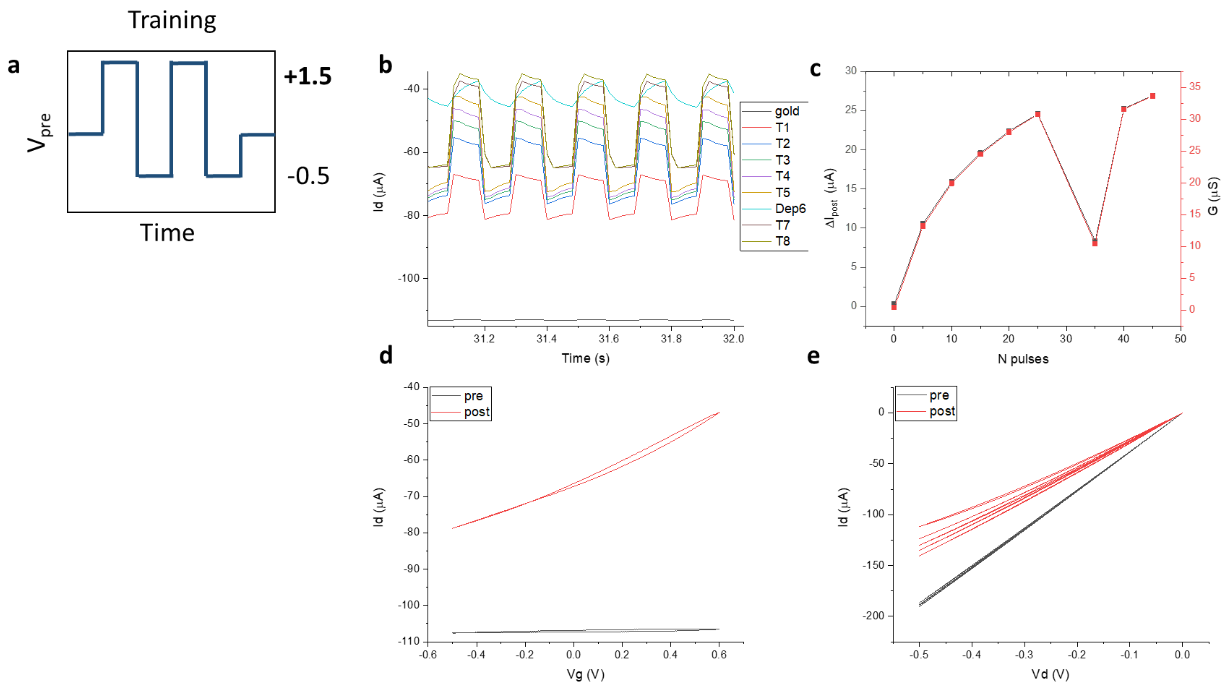

Figure S10 Voltage wave used during training experiments ($V_{pre\ max}$ = +1.5 V) (a), I_{post} vs time recorded in reading experiments (b), ΔI_{post} vs number of pulses (c), transfer curve before and after training experiments (d) and output curve recorded before and after training experiment (e).

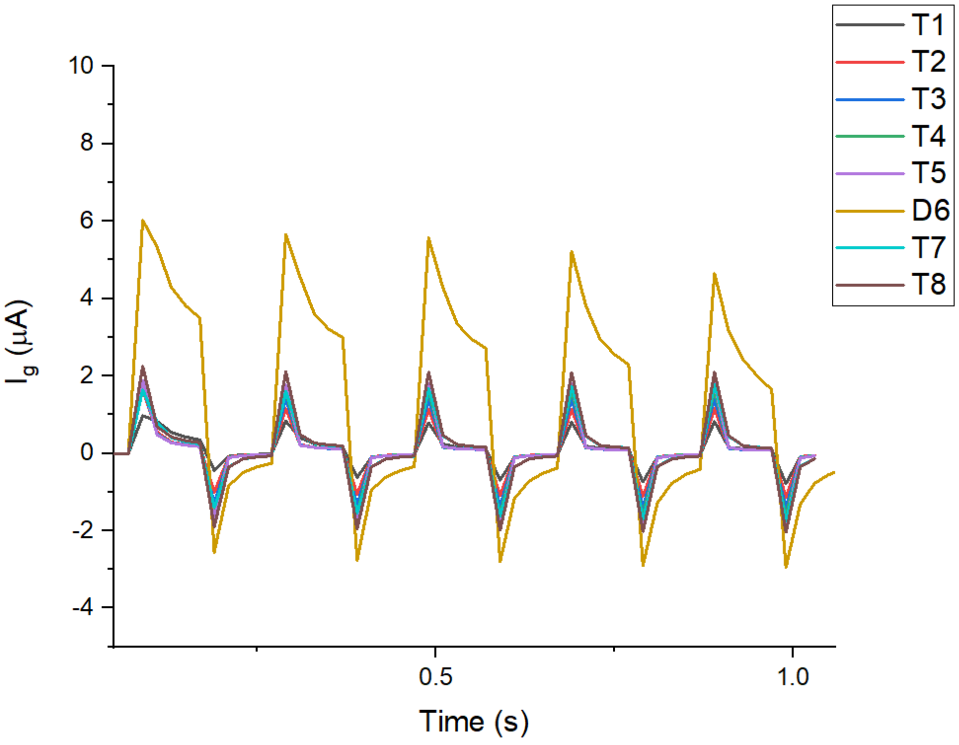

Figure S11 I_g vs time curve recorded during training cycle for V_{pre}^{MAX} equal to + 1.3 V

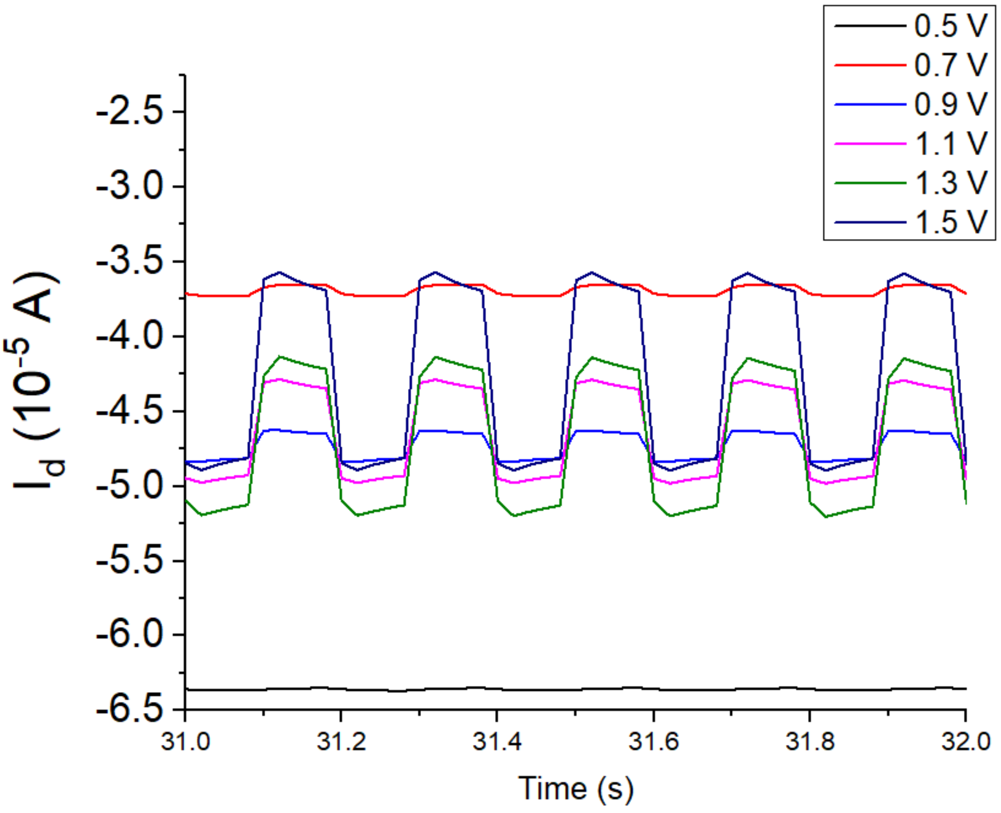

Figure S12 I_{post} vs time recorded after 25 pulses of training at different V_{pre}^{MAX}

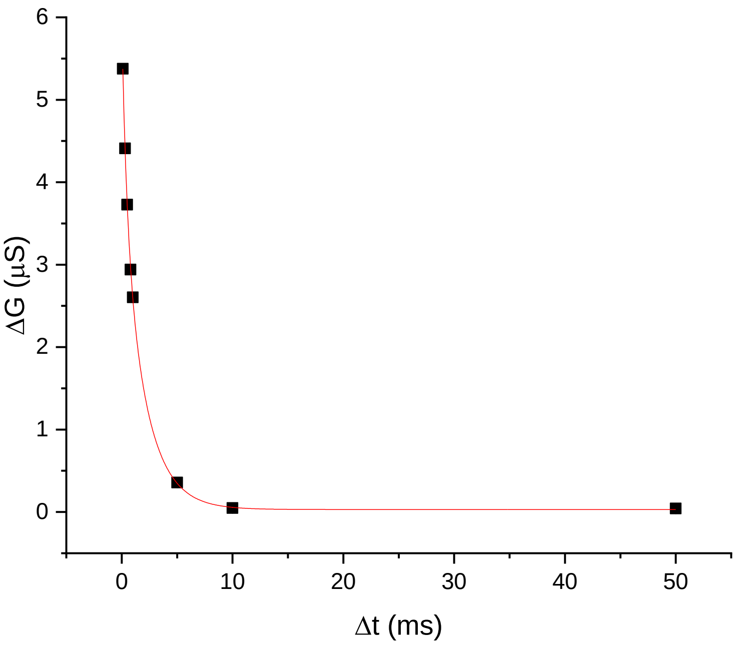

Figure S13 Exponential fit of paired pulse depotentiation experiments.

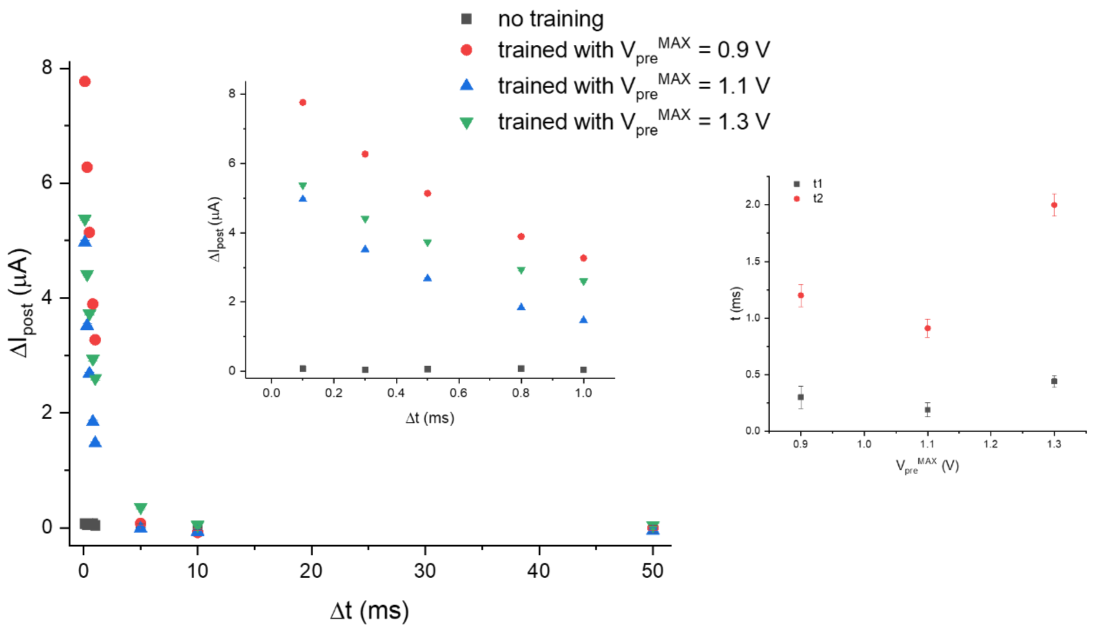

Figure S14 Exponential fit of paired pulse depotentiation experiments for OECT trained at different V_{pre}^{MAX}

function	V_{pre} (V)	N of pre synaptic pulses	N of total pre synaptic stimuli
Reading 0	- 0.3 ÷ + 0.5	160	
Training 1 **(+)**	- 0.5 ÷ V_{pre}^{MAX}	5	5
Reading 1	- 0.3 ÷ + 0.5	160	
Training 2 **(+)**	- 0.5 ÷ V_{pre}^{MAX}	5	10
Reading 2	- 0.3 ÷ + 0.5	160	
Training 3 **(+)**	- 0.5 ÷ V_{pre}^{MAX}	5	15
Reading 3	- 0.3 ÷ + 0.5	160	
Training 4 **(+)**	- 0.5 ÷ V_{pre}^{MAX}	5	20
Reading 4	- 0.3 ÷ + 0.5	160	
Training 5 **(+)**	- 0.5 ÷ V_{pre}^{MAX}	5	25
Reading 5	- 0.3 ÷ + 0.5	160	
Depot. 6 **(-)**	- 0.5 ÷ 3	10	35
Reading 6	- 0.3 ÷ + 0.5	160	
Training 7 **(+)**	- 0.5 ÷ V_{pre}^{MAX}	5	40
Reading 7	- 0.3 ÷ + 0.5	160	
Training 8 **(+)**	- 0.5 ÷ V_{pre}^{MAX}	5	45
Reading 8	- 0.3 ÷ + 0.5	160	

Table S1. Detailed procedure of training scheme

SI 1 Roles of gate and channel capacitances in LTP and STP

The working mechanisms of both STP and LTP can be better understood by drawing equivalent circuit diagrams as reported in Figure S15a. Ionic and electronic transport during the OECT operation are indicated with orange and blue lines, respectively. Given the width, length (60 × 260 µm^2) and thickness (100±10 nm) of the PEDOT:PSS layer, and the volumetric capacitance of the polymer c_v=50 F/cm^3 (https://doi.org/10.1002/adfm.201700329), we can calculate the channel capacitance $C_{ch} = W*L*t \approx 80$ nF. This value results to be significantly larger than the gate capacitance, thereby the equivalent capacitance of the OECT circuit $C_{eq} = C_g*C_{ch}/(C_g+C_{ch})$ is ultimately dominated by C_g during the entire AFM experiment (see Figure xxxb), and the variation of the OECT response induced by LTP (or LTD) is only caused by variations in C_g (Figure S15c). During LTP, the electrochemical potential drop causing the polymerization of PEDOT:PSS occurs only on the gate electrode due to its smaller capacitance and larger impedance ($Z_g \approx 2$ MΩ, $Z_{ch} \approx 0.2$ MΩ at f=10Hz). Such process involves the transfer of electrons through the Faradaic resistance R_f and leads to an increase in the gate capacitance C_g. The resulting charge transfer is compensated by the capacitive charging/discharging of the OECT channel, leading to the electrochemical de-doping of the PEDOT:PSS layer and modifying the channel resistance R_{ch}. The channel conductivity modulation is measured also during STP, but the applied gate voltage is not large enough to induce the electrodeposition of PEDOT:PSS on the gate electrode, and the C_g value remains constant.
For STP, Figure 5 highlights as the transconductance variation (ΔG) depends on the interval between consecutive pulses (Δt). As expected, there is a threshold value of Δt above which the transistor completely discharges the equivalent capacitance (channel and gate capacitances in series) between two consecutive pulses, thus showing no memory effects. From Fig. 5d it could be seen that this value is perfectly correlated with the OECT time constant, $\tau_{RC_{eq}} = R_s \cdot C_{eq}$, of 2.5ms (trained device), needed for the full charging of the OECT equivalent capacitances. In order to provide memory effect consecutive pulses need time-delays shorter than Δt, so as to have the equivalent capacitance partially charged once the second pulse starts: ΔG is more pronounced the shorter is the Δt among the first two pulses, since the capacitance has less time for discharging. This explanation is confirmed by the decrease of the Δt threshold value for OECTs having the gate in gold: Fig. S14 shows that without any LTP (no Pedot:Pss on the gate), the OECT do not exhibit STP for Δt above 0.1ms: we indeed found that the expected equivalent capacitance for the gold gated OECT leads to an OECT time constant of 0.14ms.

Fig. S15 schematically highlights the above-reported processes,working principle and the formula for the time constant calculation.

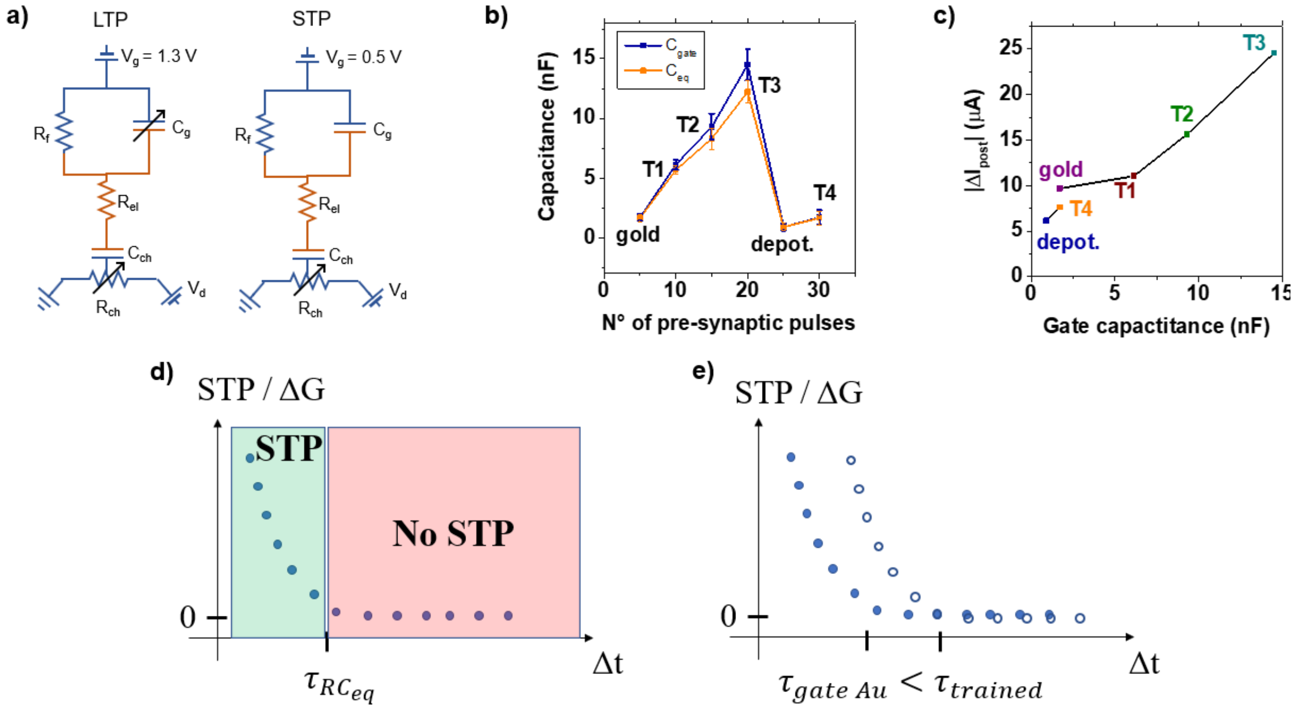

Figure S15. Role of gate and channel capacitances during STP and LTP. a) Equivalent circuit scheme for the OECT device during STP and LTP. Blue and orange lines indicate the electronic and the ionic circuit, respectively. b) Comparison between the gate capacitance and the gate/channel equivalent capacitance values during the AFM experiment. c) Effect of the gate capacitance variations on the response of the OECT device when LTP (or LTD) is performed. d) STP/transconductance variation in function of the time interval between consequent pulses, highlighting in re the region in which STP occurs.,e) Role of the training in changing the time interval region for STP, owing to the modification of the OECT ionic time response constant.
